# Management of Aesthetical and Functional Complications after Total Parotidectomy. First Long-Term Experiences with Dermal Matrix Surgimend ® in Patient Affected by Malignant Parotid Tumors

**DOI:** 10.1007/s12663-022-01761-y

**Published:** 2022-07-23

**Authors:** Paola Bonavolonta, Giorgio Iaconetta, Giovanni Improda, Cristiana Germano, Gerardo Borriello, Federica Goglia, Vincenzo Abbate, Pasquale Piombino, Luigi Califano, Giovanni Dell’Aversana Orabona

**Affiliations:** 1 Maxillofacial Surgery UnitDepartmentof Medicine and SurgeryDepartment of Neurosciences, Reproductive and Odontostomatological Sciences, University Federico II, Via Sergio Pansini 5, 80131 Naples, Italy; 2 Department of Neurosurgery, University of Salerno, Salerno, Italy; 3 Research Fellow, Department of Public Health, Federico II University of Naples, Naples, Italy

**Keywords:** Radical parotidectomy, Paralysis of the facial nerve, Sialocele, Frey Syndrome, Acellular dermal matrix, Surgimend

## Abstract

**Background:**

This is an observational cohort study on patients affected by malignant parotid tumors treated with total parotidectomy. The aim of our work is to analyze and compare the effects and complications after parotidectomy, using or not SurgiMend ®.

**Methods:**

40 patients were retrospectively enrolled between September 2014 and June 2020. Basing on the placement of SurgiMend ® for parotid lodge reconstruction, the samples were divided into two groups. Thus, the incidence rate of complications after the surgical procedure was analyzed between the two groups.

**Results:**

Patients in whom SurgiMend ® was used reported a lower rate of complications**.** The ANOVA test (*p* = 0.05) revealed a significant difference of Vancouver Scar Scale (VSS) between the two groups, representation as vascularity and pigmentation improvement, changing scar color, scar height reduction, and increased pliability.

**Conclusion:**

Although many techniques are available to fill the parotidectomy defect, improve facial contour and prevent Frey’s syndrome, the use of SurgiMend ® matrix is one of most effective and reliable method to address these complications, with the advantage of decreased operative time due to not require an additional surgical donor site.

## Introduction

Salivary gland tumors are rare and represent about < 1% of all malignant neoplasms and around 6% of Head and Neck tumors [1] [2].

The most frequent localization of salivary gland tumors (80–85%) is represented by the parotid gland, and specifically of these neoplastic diseases, 80% are benign tumors and the remaining 20% are malignant [3].

Radical parotidectomy is recommended for tumors with deep lobe involvement, suspected or confirmed high-grade tumors, or tumors with aggressive malignant potential, such as those with facial nerve involvement, multiple intraparotid masses, or cervical metastasis. In patients without facial nerve infiltration, sacrifice of parts of the nerve as radical parotidectomy does not offer better tumor control or survival advantage [4].

The main complications of parotidectomy are paresis or paralysis of the facial nerve, that can be related either by tumor infiltration or by iatrogenic damage, hypo-anesthesia of the skin, hemorrhage/hematoma, wound infection, salivary fistula, face profile asymmetry, keloids, Frey Syndrome (FS) [5, 6].

Some complications may be minimized using meticulous surgical procedures and appropriate instrumentations such as Nerve Intra-operative Monitoring (NIM) for nerve damaging prevention.

Others may be avoided using specific surgical techniques to prevent the incidence of postoperative complications like autologous tissue transplantation, which includes the use of sternocleidomastoid (SCM) muscle flap, platysma muscle flap (PMF), temporoparietal fascia (TPF), superficial musculoaponeurotic system (SMAS), buccal fat pad (BFP). Other modalities being practiced are use of non-vascularized tissues such as dermal fat graft, fat injections at subdermal layer, acellular dermal allograft and artificial material [7–15].

Autologous tissue transplantation has shown some ability to prevent infra-auricular depressed deformities and Frey’s syndrome; however, other problems exist with the use of autologous transplantation, such as additional surgical donor site trauma, increased operating time, and a limited donor quantity [16].

Therefore, acellular dermal matrix (ADM) was introduced and claims to be an ideal alternative for tissue augmentation. The advantages of ADM are that a second surgery site is not required and there is an unlimited supply of material. Acellular dermal matrix is available in abundance, is very flexible, and can easily be trimmed to a variety of shapes [16–19].

SurgiMend ® (TEI Biosciences; Boston, MA, USA) is an ADM and it has been widely used in hernia repair, muscle flap reinforcement, plastic and reconstructive surgery. It is a non-cross-linked matrix of type I and II collagen terminally sterilized with ethylene oxide and free from preservatives including polysorbate [20, 21].

We present an observational series of a cohort of patients affected by malignant parotid tumors treated with total parotidectomy. The aim of our work is to analyze and compare the effects and complications after parotidectomy, using or not SurgiMend ®.

## Materials and Methods

The data of 40 patients affected by malignant parotid tumors who underwent to total parotidectomy in our Maxillo-Facial Surgery Unit between September 2014 and June 2020 were retrospectively reviewed.

Written informed consent was obtained from all patients.

Since the retrospective nature of the study no ethical approval was required.

The demographic characteristics of the patients were collected: age, sex, medical comorbidity, risks factors (alcohol, smoke, drugs) (Table 1). Moreover, type of tumor, size and location were recorded.

**Table 1:** Demographic characteristics

	Group A 20 patients	Group B 20 patients	Total 40 patients
Age	42–75 years Mean age 58,26	44–77 years Mean age 58,13	42–77 years Mean age 58,19
Sex: Female Males	10 10	9 11	19 21

Inclusion criteria in the study were presence of parotid mass at MRI or CT scan, cytological diagnosis.

made by FNAC, confirming the presence of malignancies (Stage T1-T2 N0 M0), no previous surgical treatment.

Exclusion criteria were patients, who required concurrent surgeries: lateral neck dissection (patient cN +), full thickness skin graft, reconstruction surgery with free flaps.

Patients with advanced stage (Stage T3-T4-N +) were excluded.

Patients with history of hypersensitivity to collagen or bovine products, patients who had facial nerve sacrifice, skin or bone resection, were excluded. Additionally, patients with less than 1-month follow up were excluded from all outcome analysis.

The patients, underwent to total parotidectomy, were divided into two groups: group A and group B (each one composed by 20 patients).

Patients in group A follow by placement of SurgiMend ® in the resection site. Patients in group B without placement of an interpositional barrier.

The 20 patients of the Group B were surgically treated before the introduction of reconstruction devices such as SurgiMend ® in our clinical practice.

All the complication were evaluated during the regular follow up in both groups.

No conflict of interest is declared.

## Surgical Technique

Surgery was performed under general anesthesia with patients in supine position the head tilted to the opposite side of the lesion. We performed a modified face lift incision, which improve the aesthetic appearance of surgical scar.

Cutaneous incisions were hidden in the preauricular crease, and neck incisions were hidden in the curvilinear crease * 3 cm below the angle of the mandible. SMAS flap was raised. Superficial total parotidectomy was performed with conventional identification and preservation of the facial nerve and its branches, if it was not involved in the mass.

**Fig. 1:**
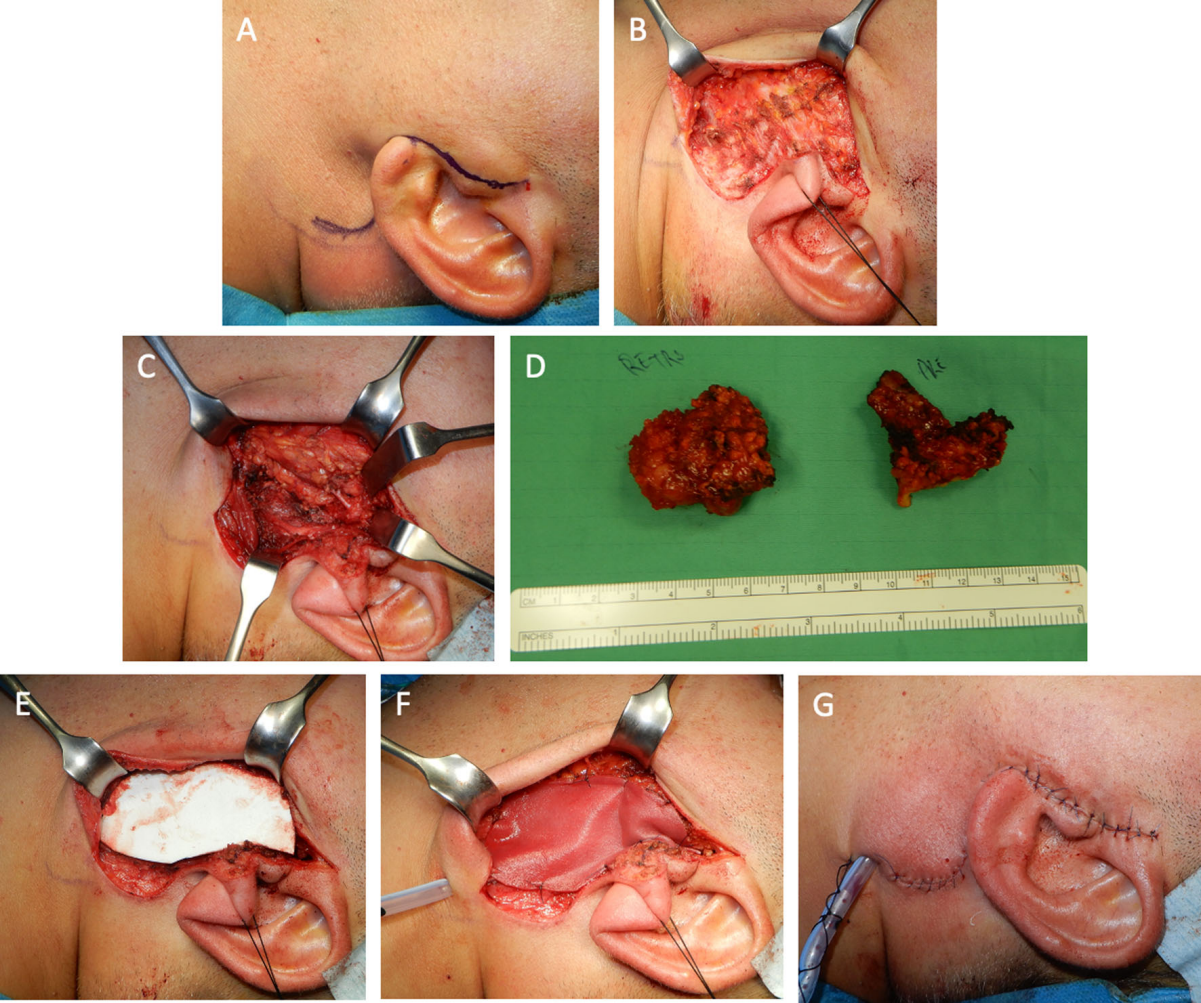
Surgical steps: **A**. Pre-operative mark, **B**. Smas incision, **C**. Superficial parotidectomy, **D**. Speciment after total parotidectomy **E**–**F**. Surgimend Schaping and Insetting. **G**. Stitched scar

Group A, after a check of the resection site in, the ADM SurgiMend® PRS (6 × 16 cm Fenestrated Semi-Oval- 1.0 mm thickness) was pre-formed in size and shape considering the dimension of the gap; then it was soaked in sterile, room temperature, 0.9% saline until hydrated (usually two minutes). Hydration gave a color change from white (dry) to pink-grey (wet). And it was carefully applied and fixed in the post-resective gap, to fill the defect that appears after parotidectomy (Fig. 1) [20].

The 20 patients of the Group B were surgically treated before the introduction of reconstruction devices such as SurgiMend ®.

Group B after performing the parotidectomy the SMAS flap was set up, this was then sutured on the zygomatic periosteum and on the parotid-masseter fascia which covers the retromandibular surgical gap [7].

Suction drains and compression bandages were applied for 1 week, and sutures were removed after 7 days in all patients of both groups.

We analyzed the incidence in the two groups of the main complications of salivary gland surgery, Frey’s syndrome, contour deformity, wound infection, seroma and Hematoma.

The cosmetic contour deformity was objectified using the Vancouver Scar Scale and a validated questionnaire, the Modified Patient Scar Assessment Questionnaire [22–25].

The follow-up period, range of 14–23 months, media of 19 months.

## Results

40 patients, 21 males and 19 females (ratio 1.10 males and 1 females); the age of the patients ranged between 42 and 77 years (mean age 58, 19), have been enrolled. (Table 1).

The characteristics of the two groups, including age, sex, tumors volume, and pathology, were homogeneous.

Post-operative histological examination is reported in Table 2.

**Table 2:** Final histology and incidence of the removed neoplasms

	Group A with *Surgimend*	Group B without *Surgimend*
Mucoepidermoid carcinoma	7 patients (35%)	6 patients (30%)
Carcinoma ex pleomorphic adenoma	3 patients (15%)	2 patients (10%)
Acinic cell carcinoma	3 patients (15%)	4 patients (20%)
Adenocarcinoma NOS	2 patients (10%)	2 patients (10%)
Adenoid cystic carcinoma	2 patients (10%)	3 patients (15%)
Squamous cell carcinoma	3 patients (15%)	2 patients (10%)
Salivary duct carcinoma	1 patient (5%)	1 patient (7%)
Undifferentiated carcinoma	1 patient (5%)	0 patients (0%)
	20	20

Complications occurred in Group A and Group B patients are reported in Table 3.

**Table 3:** Complications

Complications	Group A with *Surgimend*	Group B without *Surgimend*
Salivary Fistula	0 (0%)	2 (10%)
Frey’s Syndrome	1 (5%)	3 (15%)
Contour deformity	1 (5%)	4 (20%)
Wound Infection	0 (0%)	1 (5%)
Hematoma	2 (10%)	3 (15%)
Seroma	1 (5%)	2 (10%)
Hypertrophic scar	2 (10%)	8 (40%)
Keloid	0 (0%)	2 (10%)

Acute events were managed in the post-operative recovery time, while chronic or tardive complications were managed during the regular ambulatorial follow up.

**Fig. 2:**
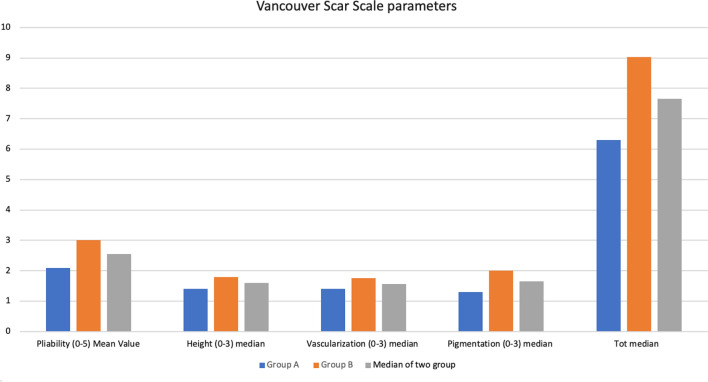
Vancouver Scar Scale parameters

Scar quality was analyzed via assessment of the Vancouver Scar Scale (VSS) score of scars at 30^th^ follow up day before patients who needed radiotherapy treatment started it. (Table 4 and Fig. 2).

**Table 4:** Vancouver Scar Scale

Pliability	Normal (0), Supple (1), Yielding (2), Firm (3), Ropes (4), Contracture (5)
Height	Flat (0), less than 2 mm (1), 2 to 5 mm (2), more than 5 mm (3)
Vascularization	Normal color (0), pink (1), red (2), purple (3)
Pigmentation	Normal (0), hypopigmentation (1), mixed (2), hyperpigmentation (3)

	Pliability (0–5) Mean Value	Height (0–3) median	Vascularization (0–3) median	Pigmentation (0–3) median	Tot median
Group A	(1–3) 1,8 ± 0.77	(1–2) 1,35 ± 0.48	(1–2) 1,3 ± 0.47	(1–3) 1,3 ± 0.57	(4–10) 5,8 ± 1.51
Group B	(1–5) 3,0 ± 1.21	(1–3) 1,85 ± 0.74	(1–3) 2 ± 0.79	(1–3) 1,95 ± 0.68	(6–11) 8,8 ± 1,93

## Discussion

Parotid gland surgery is related to various complications, some due to the pathology characteristics and some due to iatrogenic damages. Prevention is possible thanks to technology innovations, such the use of NIM, and the filling of surgical gap using ADMs or local flaps. In our study patients were divided in Group A (patients which surgery technique included the use of SurgiMend ®) and Group B (patients which surgery technique didn’t include the use of SurgiMend ®).

In group A 2 cases (10%) of hematoma, 1 case (5%) of seroma, 1 case (5%) of Frey Syndrome, 1 case (5%) of contour deformity and 2 cases (10%) of hypertrophic scar occurred. In group B 3 cases (15%) oh hematoma occurred, 4 cases (20%) of contour deformity, 2 cases (10%) of seroma, 3 cases (15%) of Frey Syndrome, 1 case (5%) of wound infection, 2 cases (10%) of salivary fistula, 8 cases (40%) of hypertrophic scar and 2 (10%) keloids.

No cases of immune rejection and abscess were highlighted in both groups.

Salivary fistula after total parotidectomy can be explained with the presence of a gland residue. Frey Syndrome in group A can be explained by displacement or unproper positioning of SurgiMend ®. We attributed the possible displacement during the first cases treated since we did not use to stitch the SurgiMend ® to surrounding tissues.

Frey Syndrome was treated during follow up by the injection of botulinum toxin.

Hematoma cases resolved in 7 days. Corticosteroids therapy was performed in these patients to reduce inflammation.

Patients who had facial contour deformities were proposed to lipofilling treatment, but no one of them accepted the treatment.

Hypertrophic scars and keloids were treated with corticosteroid infiltrations.

Wound infection was treated with antibiotic therapy. Seromas and salivary fistulas required a frequent follow up and pressuring dressings.

The study results revealed a significant difference of VSS in the two groups, representation as vascularity and pigmentation improvement, changing scar color, scar height reduction, and increased pliability. In particular we performed ANOVA test (Significatively level: *p* = 0.05) between group A and B wit *p* < 0.05 and data show a statistically significative difference among the two groups, thus validating the hypothesis that the results obtained using SurgiMend ® are better if compared to the classic procedure.

Frey's syndrome and infra-auricular depressed deformities are most common complications that can occur after total parotidectomy. For the prevention of above-mentioned two complications, a number of scholars have proposed different techniques. Sternocleidomastoid muscle flap, the superficial musculoaponeurotic system flap, temporoparietal fascia flap, the free or vascularized dermal fat graft [7–15].

Cesteleyn L et al. in their study they demonstrated that incidence of Frey’s syndrome was reduced from 33 to 4% with the use of the musculoaponeurotic layer [15].

Free fat graft as an effective reconstructive technique to prevent FS was already pointed out by other authors [11–13].

Balasundaram I et al*.* used a free fat paraumbilical graft to reconstruct the parotid bed defect. This graft is harvested through a supra/sub-umbilical or suprapubic incision. Over-correction of approximately 50% is required due to atrophy and resorption with time. However, the incidence of Frey syndrome also appeared to be reduced and reliable method of correcting facial defects after a superficial or total parotidectomy [11].

Each of these methods described seems to present some problems.

For cases treated as autologous tissue transfer, a donor site, consequent scars, double surgical access and consequent lengthening of surgical times was required.

Noteworthy is the fact that fat transplantation will be subjected to a certain degree of reabsorption and therefore the long-term postoperative results are doubtful.

To overcome the limits of these techniques, in recent times, it has been proposed its ADM devices to prevent the most common complications of parotidectomy [16].

As regards non-autologous implant there are many non-crosslinked meshes available on the market, which are derived from numerous sources (human, porcine, bovine, etc.) and tissue (dermis, intestine or bladder submucosa, pericardium, etc.) are decellularized by distinct proprietary methods, and are sterilized by one of several techniques (gamma irradiation, electron, beam irradiation, ethylene oxide, etc.) [16–19].

They are xenogeneic ADMs, inert, non-crosslinked matrices reinforce soft tissue and are a framework for cellular repopulation and neovascularization, and will support fibroblast infiltration, neovascularization [16–19].

Non-autologous implants have several advantages: an unlimited and readily available supply, ease of positioning and contouring, shorter operative times, and no donor-site morbidity. Their disadvantages are lower patient acceptability due to greater risks of infection, rejection, and/or extrusion [16–19].

Selecting the optimum matrix remains difficult, including Strattice TM (Lifecell, Branchburg, NJ, US) and SurgiMend ® TM (TEI Biosciences; Boston, MA, US) [16–19].

Adelman et al*.* in their study compared the mechanical properties of the both ADMs, Settice and SurgiMend ®, using a series of in vitro preimplantation tests. They found SurgiMend ® had increased mechanical strength compared with Strattice of equal thickness [20].

Therefore, we decided to introduce in our clinical practice the use of SurgiMend ® [20].

SurgiMend ® is an acellular dermal matrix of type I and II collagen that over the years has provided excellent results in applications such as breast surgery, burns treatment, hernia repair, muscle flap reinforcement, plastic and reconstructive surgery [20–22].

SurgiMend ® helps to prevent facial contour deformities caused by the surgical gap filling the empty space and reduce the possibility to develop seroma. Furthermore, it acts like a mechanical barrier avoiding unconventional nerve junctions, known to be the cause of Frey Syndrome.

From our study, it has emerged that the use of SurgiMend ® reduces the incidence of typical complications of parotidectomy, according to the results of the most recent literature [16].

Moreover, it improves the facial profile, reduces the infra-auricular depressed deformities and therefore the esthetic results are more satisfactory, this was objectified by a reduction in the VSS score.

VSS was first introduced in 1990 and it has been widely described in the literature. In this study, VSS was used to assess vascularity, pigmentation, thickness and pliability of hypertrophic post burn scar formation pre and post treatment to detect the difference between both groups. A limitation of our study is due to the fact that a scar scaling is used as a subjective tool, other methods described in the literature have varying degrees of reliability and validity and most of them are expensive, time-consuming, highly technological and often non-portable, making them clinically impractical. VSS is the first validated scar assessment scale and remains the most widely used scale in the clinical setting. Furthermore, a previous study found that VSS is a suitable substitute for Cutometer, Mexameter and DermaScan C, in terms of discrimination of the characteristics of the scar [23–25].

Extracellular matrix bio-scaffolds are an innovative solution to aid surgeons, improving cosmetics outcomes and reducing the rate of complication.

It has proved to be free of side effects, which makes this method indicated.

What emerged from our study is that in the group of patients with the SurgiMend ® we found a lower score for each parameter, which corresponds to a greater degree of satisfaction of the wounds, better perceived appearance and a better consciousness of these. Results were confirmed by the ANOVA test; indeed a statistically significant difference between group A and B emerged from the test, using a *p*_value_ < 0.05.

## Conclusion

Parotid surgery is a technically challenging surgery due to the in important structures in the vicinity.

Many complications may occur either during and after surgery among them the most important are Frey’s syndrome and infra-auricular depressed deformities.

Although many techniques are available to fill the parotidectomy defect, improve facial contour and prevent Frey’s syndrome, the use of SurgiMend ® matrix is one of most effective and reliable method to address these complications, with the advantage of decreased operative time due to not require an additional surgical donor site.

Further studies are needed to state a long-term durability of these novel meshes, but in the short term, the application of SurgiMend ® matrix, no crosslinked biologic material have shown to bring important advantages such as no immune rejection, low risk of infections, hematoma or salivary fistula.
